# Granulomatous lobular mastitis: diagnostic and prognostic biomarkers

**DOI:** 10.3389/fimmu.2026.1960862

**Published:** 2026-10-22

**Authors:** Xi Yang, Jingwei Li, Hongyi Liang

**Affiliations:** 1 First Clinical Medical College, Shandong University of Traditional Chinese Medicine, Jinan, Shandong, China; 2 Breast and Thyroid Surgery, Affiliated Hospital of Shandong University of Traditional Chinese Medicine, Jinan, Shandong, China

**Keywords:** granulomatous lobular mastitis, biomarkers, diagnostic, prognostic, treatment

## Abstract

Granulomatous lobular mastitis (GLM) is a chronic, immune-mediated inflammatory breast disease of elusive etiology that presents formidable challenges in clinical management. This clinically oriented review synthesizes current evidence on biomarkers according to their potential applications in GLM diagnosis, differential diagnosis, disease-activity assessment, abscess prediction, recurrence surveillance, and treatment guidance. We critically evaluate conventional inflammatory indices, endocrine markers, immune-cell phenotypes, cytokines, complement-related markers, and emerging molecular signatures, while considering the influence of specimen type, disease stage, comparator population, and assay methodology on reported findings. Recent single-cell transcriptomic and metabolomic studies have expanded the biomarker landscape beyond circulating proteins, whereas epitranscriptomic regulation and immune-cell metabolic reprogramming remain emerging areas with limited direct GLM-specific evidence. Overall, routinely available markers such as CRP, ESR, NLR, SII, and prolactin currently offer the greatest clinical accessibility but limited disease specificity, whereas novel immune and molecular biomarkers remain largely exploratory. Future multicenter prospective studies integrating clinical, imaging, and molecular data are needed to develop robust biomarker panels and support precision management of GLM.

## 1 Introduction

Granulomatous lobular mastitis (GLM) is characterized histologically by non-necrotizing granulomatous inflammation centered on the terminal duct lobular unit of the breast (1), which was first described by Kessler and Wolloch in 1972 (2). Granulomatous mastitis (GM) is a broader descriptive term for granulomatous inflammation of the breast. GLM denotes a lobule-centered histopathological pattern, whereas idiopathic granulomatous mastitis (IGM) denotes disease diagnosed after exclusion of identifiable causes (3, 4). GLM is the principal focus of this review. GM and IGM are retained when describing studies that used those designations.

GLM predominantly affects parous women of reproductive age, particularly those with a recent history of pregnancy and lactation (5, 6). Clinically, GLM typically manifests as a painful breast mass in the peripheral breast, often accompanied by pain and local skin erythema. The disease may progress to form sinus tracts and fistulas, and some patients present with systemic symptoms such as erythema nodosum of the limbs and fever. Importantly, GLM may follow a prolonged and relapsing course, creating substantial challenges for diagnosis, disease monitoring, and long-term management (7).

The pathogenesis of GLM is considered multifactorial and may involve organ-specific autoimmune responses, endocrine dysregulation, microbial stimuli, oxidative stress, and tissue injury (8–10). Hyperprolactinemia has been proposed to contribute to chronic inflammation by disrupting the Treg/Th17 balance (11). Specifically, *Corynebacterium parakroppenstedtii* triggers local tissue inflammation by secreting corynekropbactins that chelate host iron, induce mammary epithelial cell death, and upregulate pro-inflammatory cytokines such as IL-6 (12). Oxidative stress and reactive oxygen species may further contribute to persistent tissue injury and inflammatory signaling (13). A history of inverted nipples has been associated with recurrence and new occurrence of GLM after clinical recovery, although this observational association does not establish a causal role in initial disease onset (14). Trauma may initiate the inflammatory cascade as a non-specific stimulus (15). These factors may interact with local and systemic immune dysregulation to establish a self-sustaining inflammatory microenvironment.

Despite increasing recognition of its immune-mediated nature, the clinical management of GLM remains challenging (16, 17). Diagnosis currently relies mainly on clinical presentation, imaging, and histopathological examination, while conventional laboratory indicators such as inflammatory indices and endocrine parameters provide supportive but generally nonspecific information (3). Recent consensus recommendations have further emphasized standardized clinical classification and pathological confirmation as important foundations for diagnosis and longitudinal assessment in GLM (18). These markers may reflect the systemic inflammatory burden but often provide limited information regarding disease stage, abscess formation, treatment response, or recurrence risk. Consequently, there is a growing need for biomarkers that can complement conventional diagnostic approaches and provide clinically useful information throughout the disease course, from initial diagnosis and differential diagnosis to dynamic disease monitoring and recurrence prediction.

Previous reviews have primarily focused on the etiology, immunopathogenesis, treatment, or overall characteristics of the evidence base in granulomatous mastitis (5, 6, 8, 17). In contrast, an integrated assessment that maps candidate biomarkers to specific clinical decision points remains limited. The present review therefore adopts a clinically oriented framework that evaluates biomarkers according to their potential roles in diagnosis, differential diagnosis, disease-activity assessment, abscess prediction, recurrence surveillance, and treatment guidance. In addition to reported biomarker alterations, we explicitly consider biological specimen, comparator population, disease stage, analytical methodology, and translational readiness when interpreting the evidence. By distinguishing routinely accessible clinical markers from emerging molecular signatures, this framework is intended to bridge biomarker discovery with clinical decision-making in GLM.

This review integrates clinical, pathological, immunological, and emerging molecular evidence to characterize the current biomarker landscape of GLM. Rather than focusing solely on whether individual biomarkers are increased or decreased, we evaluate their potential clinical utility according to specific clinical questions, including diagnosis, differential diagnosis, disease activity assessment, abscess prediction, recurrence surveillance, and therapeutic stratification. We also consider the biological context of biomarker findings, including the sample source, disease stage, comparator population, and analytical method, to explain inconsistencies across studies. Finally, emerging evidence from single-cell transcriptomics and metabolomic profiling is incorporated, while epitranscriptomic regulation and immune-cell metabolic reprogramming are considered emerging research directions with limited direct GLM-specific evidence. Recent evidence has further strengthened the autoimmune framework of GLM by integrating cytokine dysregulation, T-cell imbalance, and single-cell transcriptomic findings (8).

## 2 Diagnostic biomarkers

### 2.1 Peripheral blood inflammatory indices

#### 2.1.1 CRP, ESR

C-reactive protein (CRP) and erythrocyte sedimentation rate (ESR) are widely available indicators of systemic inflammatory burden in GLM (19, 20). During the active phase of the disease, both CRP and ESR are generally increased (21), with CRP levels showing a positive correlation with the degree of focal inflammation and the presence of erythema nodosum (22, 23). Changes in circulating neutrophil, platelet, and lymphocyte counts further reflect systemic inflammatory activity. From a clinical perspective, these markers are useful as readily accessible adjunctive indicators for assessing inflammatory severity and monitoring disease activity. However, their limited specificity prevents them from reliably distinguishing GLM from other inflammatory or malignant breast disorders when used alone. Their greatest clinical value is therefore likely to lie in combination with disease-specific immune or molecular biomarkers.

#### 2.1.2 NLR, PLR, SII

Because individual inflammatory indices have limited specificity, composite hematological indices may provide a more integrated representation of systemic immune-inflammatory status. The neutrophil-to-lymphocyte ratio (NLR), platelet-to-lymphocyte ratio (PLR), and systemic immune-inflammation index (SII) are readily calculated from routine blood counts and therefore have practical advantages in resource-limited clinical settings (24–26). In GLM, elevated NLR and SII have been associated with greater disease activity, while NLR has also shown prognostic value for recurrence. These indices may therefore serve as inexpensive adjunctive tools for initial inflammatory assessment, longitudinal monitoring, and risk stratification. Nevertheless, because NLR, PLR, and SII are influenced by infection, systemic inflammation, medication, and other comorbid conditions, they should be interpreted as nonspecific indicators and preferably integrated with clinical, imaging, and disease-specific biomarkers.

#### 2.1.3 Iron-related biomarkers

Iron-related biomarkers have been reported in GLM. Liu et al. found that seven of 10 patients with GLM and confirmed *Corynebacterium kroppenstedtii*-like strain infection had serum iron levels below the normal range, while experimental infection with *C. parakroppenstedtii* reduced serum iron and transferrin saturation in rats (12). Another clinical study also reported lower serum iron levels in the abscess stage than in the mass and remission stages (27). Although serum iron may provide complementary information on disease phenotype, its diagnostic and prognostic value remains insufficiently validated.

### 2.2 Prolactin

Serum prolactin (PRL) has attracted increasing clinical interest as an endocrine biomarker in GLM. Abnormal PRL levels have been associated with greater lesion burden and treatment outcomes, while longitudinal changes in PRL have also been associated with recurrence risk (28, 29). Mechanistically, PRL/PRLR signaling has been proposed to interact with JAK/STAT-related and NF-κB-related inflammatory pathways and may influence T-cell immune balance (8). However, these mechanistic associations should be distinguished from the clinical utility of circulating PRL as a biomarker.

### 2.3 Innate immune markers

#### 2.3.1 Toll-like receptors

Pattern-recognition receptors may contribute to the initiation and amplification of innate immune responses in GLM. Lipid components exuded from mammary ducts act as endogenous damage-associated molecular patterns (DAMPs), which, together with exogenous pathogen-associated molecular patterns (PAMPs) derived from *Corynebacterium* species that may colonize the local tissue (30), activate pattern recognition receptors highly expressed in the lesional tissue. TLR2 and TLR4 are overexpressed in acute-phase GLM lesions, with expression levels significantly higher in the acute phase than in the subacute and chronic phases (31). Serum TLR3 is downregulated in GLM patients, and this downregulation may be associated with decreased levels of LL-37 and IL-36α (32).

#### 2.3.2 Macrophage phenotypic markers

Macrophage heterogeneity is an important component of the inflammatory microenvironment in GLM. Studies have also demonstrated alterations in macrophage-associated markers such as CD86 and CD206 in GLM lesions (33). Single-cell RNA sequencing has further revealed inflammatory macrophage populations with heterogeneous inflammatory transcriptional profiles in GLM lesions. Inflammatory macrophage populations showed altered expression of genes involved in Fc-receptor signaling, NADPH oxidase activity, cytokine signaling, and phagocytosis, including *FCGR1A, CYBB, NCF1, and TNFSF10* (9, 34). These findings support a role for macrophage-associated inflammatory programs in GLM, but their value as clinically measurable biomarkers has not yet been established.

Beyond cellular phenotype, macrophage-related molecules may have direct biomarker potential. Karatas et al. reported lower serum ISM1 levels in 30 patients with IGM than in 30 controls undergoing breast reduction surgery (35). This case–control comparison does not establish its ability to distinguish IGM from breast cancer or other inflammatory breast diseases, and the reported diagnostic performance has not been independently validated (**Table 1**). Experimental studies in other inflammatory conditions suggest that ISM1 may influence macrophage efferocytosis and inflammatory resolution (36), but these mechanisms have not been directly demonstrated in GLM. Aydın et al. reported lower serum galectin-3, LL-37, IL-36α, and TLR3 levels in patients with IGM than in healthy controls (32). These observational findings do not establish impaired macrophage function or a causal contribution to persistent inflammation.

**Table 1:** Appraisal of selected diagnostic and prognostic biomarkers and models.

Marker/model	Sample size/events	Comparator	Performance	Validation/limitation	Study design	Ref.
Fib/Alb	55 IGM, 50 BC, 50 HC	BC	AUC=0.792	No independent validation	Single-center retrospective comparative study	(60)
NLR	41 IGM,8 recurrences	Nonrecurrence	Recurrence prediction: AUC=0.76 (cutoff 5.02)	No independent validation	Retrospective cohort at 2 centers	(72)
SII	245 GM, 56 recurrences	SII >836 vs ≤836 for recurrence	Recurrence: unadjusted HR = 2.873 (95% CI 1.624–5.084)	No independent validation	Surgical cohort	(24)
AGR	85 IGM, 16 recurrences	Nonrecurrence	Recurrence prediction: AUC=0.869 (cutoff ≤1.179)	No independent validation and few recurrence events	Single-center retrospective cohort	(73)
IL-33	25 IGM, 28 BC, 25 HC	BC vs IGM	No significant between-group difference (P = 0.43)	Diagnostic accuracy not assessed	Single-center descriptive-analytical study	(70)
ISM1	30 IGM, 30 controls	Breast-reduction controls	AUC=0.768	No disease controls or independent validation	Case–control study	(35)
Defensins	50 IGM, 50 BC, 50 HC	BC vs IGM	BC vs IGM: AUCs = 0.975, 0.948 and 0.956 for α-defensin 1, β-defensin 1 and β-defensin 2, respectively (**Figure 2** in ref. 69)	No independent validation; excluded from the final stepwise model	Single-center cross-sectional study	(69)
miR-451a + miR-5571-3p	55 GLM, 55 BC, 55 FA	Pooled BC & FA	Apparent AUC = 1.00	Mass-stage GLM only without independent validation	Single-center cross-sectional diagnostic study	(63)
Fecal butanoic acid	35 GLM, 26 HC	HC	AUC=0.66	No independent validation	Prospectively recruited case–control comparison	(58)
Fecal isohexanoic acid	34 GLM, 5 recurrences	Nonrecurrence	One-year recurrence prediction: AUC = 0.917	No independent validation and only five recurrences	One-year follow-up cohort	(58)
Hematological model	Development 120, validation 20	PDM	Internal test AUC = 0.807; external validation AUC = 0.750	Small validation cohort from another hospital campus	Retrospective case–control study	(71)
Neural network	212 IGM, 37 recurrences	Nonrecurrence	Recurrence prediction: training/validation/test AUCs = 0.938/0.880/0.913	Internal validation with few recurrence events	Single-center retrospective cohort	(76)

Markers are ordered by type: routine hematological and serum protein indices, cytokines, other immune-related proteins, circulating RNA markers, microbiota-derived metabolites, and multivariable models. Event counts indicate recurrence. GM, granulomatous mastitis; GLM, granulomatous lobular mastitis; IGM, idiopathic granulomatous mastitis; HC, healthy controls; BC, breast cancer; FA, fibroadenoma; PDM, periductal mastitis; Fib/Alb, fibrinogen-to-albumin ratio; NLR, neutrophil-to-lymphocyte ratio; SII, systemic immune-inflammation index; AGR, albumin-to-globulin ratio; ISM1, isthmin-1; AUC, area under the receiver operating characteristic curve; HR, hazard ratio. SII threshold unit: ×10^9^/L. The final CA15^-3^ + CA125 model achieved an AUC of 0.83, defensins were excluded. Development: 60 GLM and 60 PDM; additional validation: 6 GLM and 14 PDM. AUCs refer to the internal test and additional validation cohorts, respectively. Training/validation/test: 148/32/32 patients and 25/6/6 recurrence events; AUCs follow the same order. The hematological model was validated at another campus of the same hospital.

#### 2.3.3 Complement system

Alterations in complement components have been reported in GLM, suggesting involvement of complement activation in the inflammatory response. In a case-control study of 107 patients with GLM and 73 patients with fibroadenoma, Xie et al. found significantly higher serum C3 and C4 levels in the GLM group (P < 0.001), and C3 remained independently associated with GLM in multivariable logistic regression analysis (33). Complement activation has also been demonstrated in lesional tissue and circulating exosomes. Li et al. reported increased C3/C3a–C3aR and C5/C5a–C5aR1 signaling in lesional tissues from patients described as having GM, together with enrichment of C3a and C5a in circulating exosomes, whereas peripheral blood C3 and C5 levels did not differ significantly (37). These findings suggest compartment-dependent activation of the complement system in GLM. However, the diagnostic and prognostic performance of individual complement components remains insufficiently validated.

#### 2.3.4 Inflammasome and pyroptosis

NLRP3 inflammasome activation promotes caspase-1-dependent maturation of IL-1β and IL-18 and induces pyroptosis through cleavage of gasdermin D (GSDMD) (38). The study by Zuo et al. (39) demonstrated increased expression of cleaved caspase-1 and GSDMD-N in GLM lesional tissues, with typical pyroptotic morphological changes observable under transmission electron microscopy. These findings support the involvement of inflammasome-mediated pyroptotic injury in the local inflammatory microenvironment and suggest a possible contribution to neutrophil-rich inflammation and tissue damage. However, these molecules are currently better regarded as mechanistic or candidate pharmacodynamic biomarkers than as clinically validated circulating biomarkers.

#### 2.3.5 Neutrophils and neutrophil extracellular traps

Neutrophils are the first immune cells to respond to chemotactic signals, clearing foreign substances through phagocytosis, degranulation, and release of neutrophil extracellular traps (NETs) (40). In GLM lesions, macrophages and neutrophils abundantly secrete S100A8/S100A9, which further recruits more neutrophils to breast tissue via the TLR4/NF-κB pathway. Neutrophil-released NETs directly disrupt mammary lobular architecture, and myeloperoxidase (MPO) and proteases within NETs activate macrophages to release IL-1β and TNF-α, further stimulating S100A8/S100A9 production. Furthermore, NET fragments may act as autoantigens, potentially triggering autoimmunity (41). Serum soluble triggering receptor expressed on myeloid cells-1 (sTREM-1) has also been investigated. Ates et al. reported higher serum sTREM-1 levels in patients with IGM than in controls, including patients in remission who were not receiving corticosteroids (42). These findings suggest a possible association with persistent inflammatory activity, but its utility for longitudinal monitoring or predicting treatment response remains unvalidated.

#### 2.3.6 NK cell phenotypic markers

Natural killer (NK) cells also contribute to immune dysregulation in GLM. Peripheral blood CD16+CD56+ NK cells have been reported to increase during active disease, while CD57+ NK-cell populations may peak during the subacute phase (43, 44). These temporal patterns raise the possibility that NK-cell subsets may provide stage-related immunological information. However, their diagnostic and prognostic value remains exploratory and requires validation against clinically defined disease stages and relevant control populations.

The proposed innate and endocrine inflammatory mechanisms involved in GLM are summarized in **Figure 1**.

**Figure 1:**
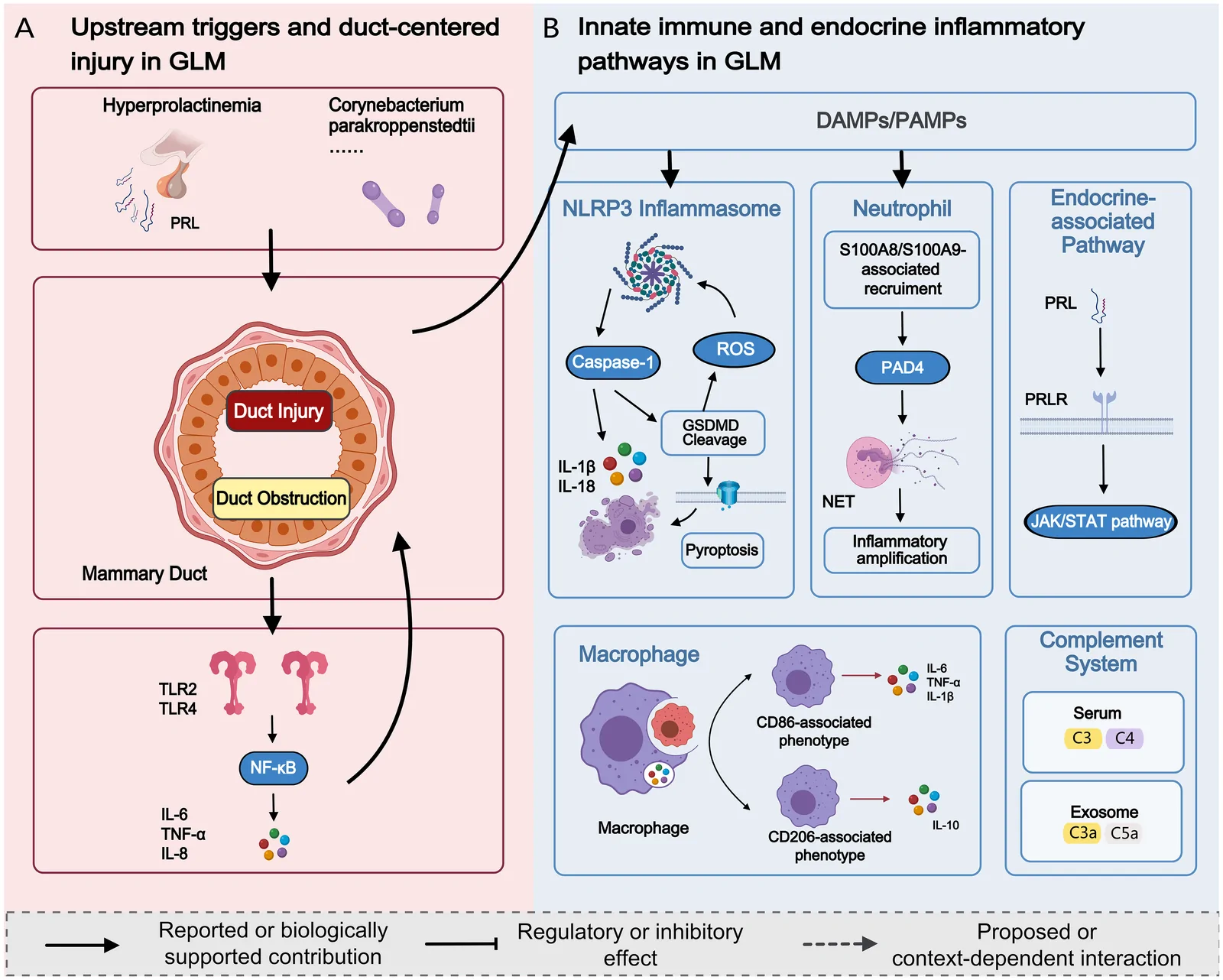
Innate and endocrine inflammatory mechanisms in granulomatous lobular mastitis (GLM). Created with MedPeer (medpeer.cn). **(A)** Proposed upstream triggers of GLM include hyperprolactinemia and microbial factors such as *Corynebacterium* species, which may contribute to duct obstruction, duct-centered epithelial injury, and activation of innate inflammatory signaling. Duct injury is associated with TLR2/TLR4-related NF-κB activation and increased production of inflammatory mediators including IL-6, TNF-α, and IL-8. **(B)** Duct injury-associated DAMPs/PAMPs may amplify local inflammation through several interconnected pathways. NLRP3 inflammasome activation promotes caspase-1-mediated maturation of IL-1β and IL-18 and induces GSDMD-dependent pyroptosis. S100A8/S100A9-associated signaling has been implicated in neutrophil recruitment, PAD4-dependent neutrophil extracellular trap (NET) formation, and neutrophil-rich tissue injury. Prolactin-associated signaling through PRLR and JAK/STAT may further modulate the inflammatory microenvironment. Complement-related alterations, including increased serum C3/C4 and exosome-associated C3a/C5a, support the presence of compartment-dependent innate immune activation. Macrophage phenotypic heterogeneity may also contribute to inflammatory amplification and tissue injury. Solid arrows indicate reported or biologically supported contributions; dashed arrows indicate proposed or context-dependent interactions; blunt-ended lines indicate regulatory or inhibitory effects.

### 2.4 Adaptive immune markers

#### 2.4.1 T-cell polarization and CD4^+^/CD8^+^ ratio

Alterations in T-cell subsets have been reported in both peripheral blood and lesional tissue in GLM, supporting the involvement of adaptive cellular immunity in disease pathogenesis (44–46). Stage-dependent changes in the CD4^+^/CD8^+^ ratio may reflect alterations in cellular immune balance during disease evolution. Yu et al. reported stage-dependent changes in the CD4^+^/CD8^+^ ratio in GLM lesions, suggesting that the balance between CD4^+^ and CD8^+^ T-cell populations may change during disease evolution (47). However, the diagnostic or disease-monitoring value of the CD4^+^/CD8^+^ ratio has not yet been independently validated.

#### 2.4.2 Treg/Th17 imbalance

The Treg/Th17 axis has been proposed as an important component of adaptive immune dysregulation in GLM (8). Several studies have reported abnormalities in Treg abundance, FOXP3 expression, or suppressive function, together with increased Th17-cell activity and RORγt expression. However, the direction of Treg alterations is not entirely consistent across studies (45, 46). For example, activated Treg populations and FOXP3 expression may increase during active disease, whereas specific CD4^+^CD25^+^CD127^low/negative^ Treg subsets have been reported to be reduced compared with healthy controls (48). This apparent discrepancy may reflect differences in Treg subset definitions, phenotypic markers, functional states, sample sources, and disease stage. Therefore, Treg should not be considered a single homogeneous biomarker. More detailed immunophenotyping and single-cell approaches may help determine which Treg/Th17 signatures are most relevant to disease activity, abscess formation, and recurrence.

#### 2.4.3 Th1/Th2 imbalance

Th1/Th2 balance deviation also represents a significant dimension of immune dysregulation in GLM. Increased Th2-associated cytokines, including IL-4 and IL-5, together with relatively reduced IFN-γ, have been reported in some clinical studies (49). Such changes may contribute to persistence of inflammation and incomplete resolution. From a biomarker perspective, however, individual Th1- or Th2-associated cytokines are unlikely to provide sufficient specificity, and their clinical interpretation should take disease stage and the broader cytokine network into account.

#### 2.4.4 Immunoglobulins

Humoral immune alterations have also been reported in GLM, although findings vary according to immunoglobulin isotype, comparator population, and clinical phenotype. Serum IgA has been reported to be significantly increased in GLM compared with healthy controls (22), while IgE was higher in GLM than in fibroadenoma in a recent case-control study (33). IgG4 expression was generally low in GLM tissues, although Kong et al. reported higher expression in patients without nipple retraction than in those with nipple retraction (43). These findings suggest that immunoglobulin profiles may reflect humoral immune activity or phenotypic heterogeneity, but their diagnostic value remains limited and requires further validation.

#### 2.4.5 Autoantibodies

The clinical relevance of autoantibodies in GLM remains controversial. Li et al. reported higher levels of anti-HIS, anti-Ro52, and anti-dsDNA in patients with GLM than in healthy controls (22), whereas another clinical study reported low positivity rates for conventional autoantibodies in patients with IGM (50). These findings suggest that although GLM may share selected autoimmune features, it does not consistently exhibit the serological pattern of a classical systemic autoimmune disease. Therefore, routine autoantibody testing should not currently be regarded as a standalone diagnostic biomarker for GLM, although selected antibody profiles may provide mechanistic information in research settings.

Adaptive immune dysregulation and cellular crosstalk in GLM are summarized in **Figure 2**.

**Figure 2:**
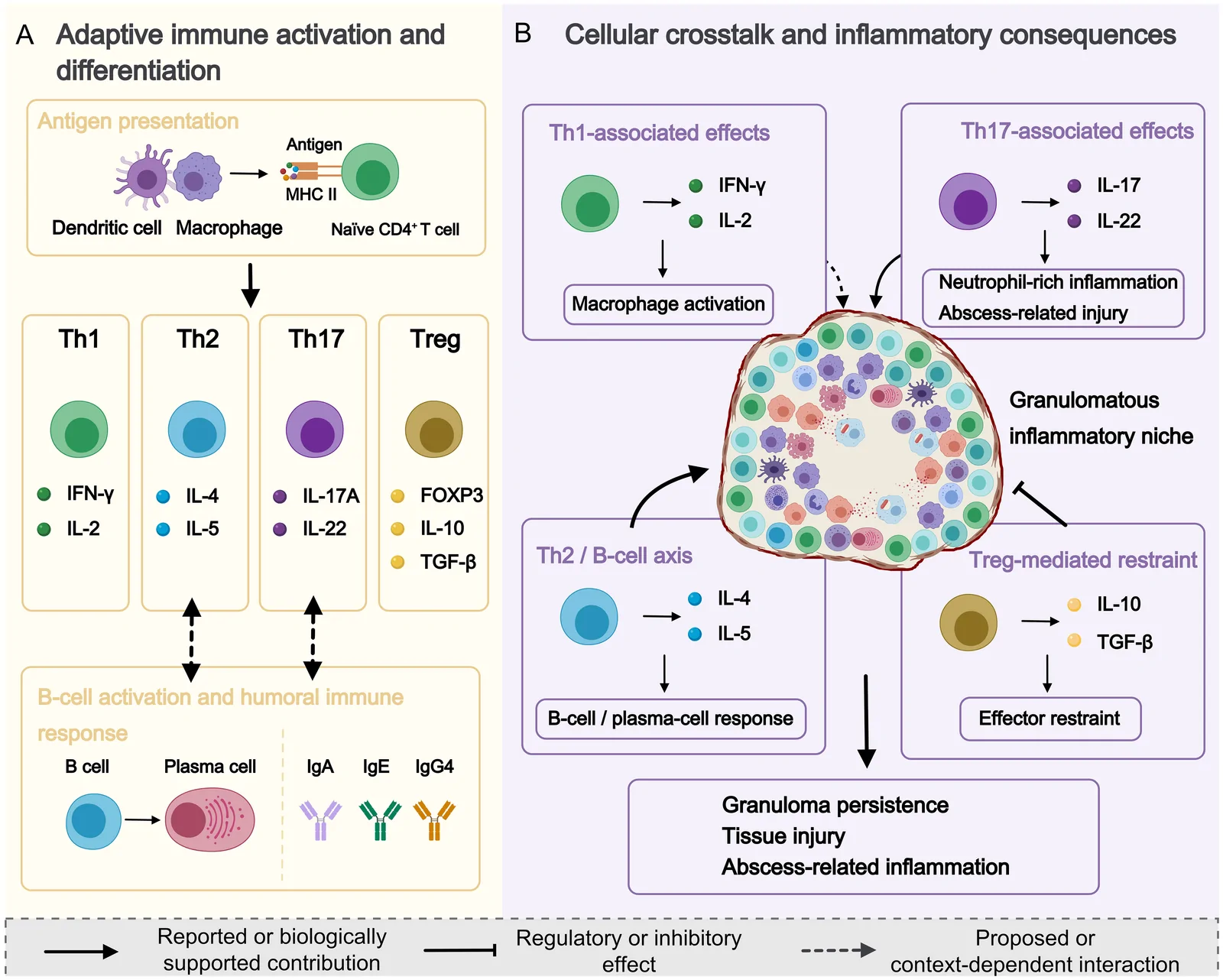
Adaptive immune dysregulation and cellular crosstalk in granulomatous lobular mastitis (GLM). Created with MedPeer (medpeer.cn). **(A)** Adaptive immune activation begins with antigen presentation by antigen-presenting cells, including dendritic cells and macrophages, to naïve CD4+ T cells through MHC II. Subsequent differentiation gives rise to Th1-, Th2-, Th17-, and regulatory T-cell-associated responses. Th1-associated mediators include IFN-γ and IL-2, Th2-associated mediators include IL-4 and IL-5, Th17-associated responses include IL-17 and IL-22, and Treg-associated markers include FOXP3, IL-10, and TGF-β. B-cell activation and plasma-cell differentiation may contribute to humoral immune alterations, including changes in IgA, IgE, and IgG4. **(B)** These adaptive immune components interact within the granulomatous inflammatory niche. Th1-associated signaling may promote macrophage inflammatory activity, whereas Th17-associated signaling may contribute to neutrophil-rich inflammation and abscess-related tissue injury. The Th2/B-cell axis may support humoral immune responses, while Treg-associated pathways may restrain excessive effector responses. Together, these interactions may contribute to granuloma persistence, tissue injury, and abscess-related inflammation. Solid arrows indicate reported activation or contribution, blunt-ended lines indicate inhibition or negative regulation, and dashed arrows indicate proposed or context-dependent interactions.

### 2.5 Cytokine networks

Available evidence suggests that GLM involves dysregulation of an interconnected cytokine network rather than an isolated abnormality in a single cytokine.

Among pro-inflammatory mediators, IL-6 is one of the most extensively investigated cytokines in GLM and has been reported to increase in circulating blood and lesional tissue, with higher levels associated with greater inflammatory activity (21, 22). TNF-α and IL-8 have also been reported to increase in some clinical cohorts, whereas IL-1β is closely associated with inflammasome activation and abscess-related inflammatory injury (22, 51, 52). Collectively, these mediators may participate in interconnected inflammatory pathways involving NF-κB activation, macrophage–neutrophil crosstalk, and inflammasome signaling rather than acting as independent disease-specific biomarkers.

IL-17-related findings illustrate the context dependence of cytokine biomarkers in GLM. Several clinical studies have reported increased circulating IL-17 or IL-17A levels in patients with IGM (21, 51), whereas other cohorts found no statistically significant difference compared with healthy controls. Saydam et al. additionally reported increased IL-22 and IL-23, supporting activation of the IL-23–Th17 axis despite the lack of a significant difference in circulating IL-17 in their cohort (53). Importantly, recent tissue-based evidence showed significantly higher IL-17 expression in GM lesions than in normal breast tissue adjacent to benign breast lesions, with positive expression increasing from the mass stage to the abscess and post-ulcerative stages (31.82%, 65.00%, and 72.22%, respectively, P = 0.021) (54). These discrepancies may reflect differences in cytokine isoforms, biological compartments, disease stage, sample size, assay methodology, treatment status, and patient characteristics. Moreover, circulating IL-17 concentrations may not directly reflect local Th17-cell activity within lesional tissue. Current evidence therefore supports involvement of the Th17 axis in GLM, but the value of circulating IL-17 as a standalone diagnostic or prognostic biomarker remains uncertain.

Koksal et al. reported higher serum IL-10 levels in patients with IGM than in healthy controls, including significantly higher levels in the remission subgroup (51). In a separate GLM cohort, IL-10 levels were higher in patients with abscess formation than in those without abscess formation (55). In contrast, another cohort observed numerically lower IL-10 levels during active disease than during remission, although the difference did not reach statistical significance (56). These findings suggest that circulating IL-10 may reflect the dynamic regulatory response accompanying different inflammatory states.

Findings for Th1- and Th2-associated cytokines are similarly heterogeneous. Reported IFN-γ and IL-2 changes vary according to biological compartment and study cohort, with both increased and decreased expression reported across blood, serum, and tissue samples. IL-4 also appears to vary according to clinical context. Du et al. reported lower serum IL-4 levels in patients with abscess formation than in those without abscess formation (55), while stage-dependent changes have been described in other cohorts (49). Yang et al. evaluated IL-2 and IL-4 expression in pathological tissues from patients with granulomatous mastitis at different clinical stages and reported stage-dependent alterations in both cytokines (57). These findings suggest that cytokine profiles may reflect disease evolution; however, their value as clinically applicable biomarkers remains uncertain due to limited validation.

Overall, cytokine findings in GLM remain heterogeneous across different biological specimens, disease stages, and study populations. Such variability limits the clinical interpretation of individual cytokines as standalone biomarkers. A model incorporating neutrophil count, ESR, IL-4, IL-10, and IFN-α has shown potential for distinguishing patients with and without abscess-forming GLM, but prospective prediction of incident abscess formation remains unestablished (55).

The clinical applications and reported alterations of cytokine biomarkers are summarized in **Supplementary Table 1**.

### 2.6 Emerging cell-state and molecular biomarkers

Recent single-cell studies have expanded GLM biomarker research beyond bulk protein measurements by identifying disease-associated cell states and transcriptional signatures. Single-cell RNA sequencing of GLM lesions has revealed marked immune-cell heterogeneity, including increased macrophage and neutrophil infiltration and distinct inflammatory macrophage populations, with altered expression of genes involved in Fc-receptor signaling, NADPH oxidase activity, cytokine signaling, and phagocytosis (9, 34). These findings provide a molecular basis for identifying cell-state-specific candidate biomarkers. However, current single-cell studies have involved small numbers of patients and have not established diagnostic thresholds or independently validated associations with clinically relevant outcomes. Thus, single-cell signatures should currently be regarded primarily as a biomarker-discovery resource rather than clinically applicable diagnostic or prognostic tests.

Metabolomic profiling provides another potential source of clinically measurable biomarkers. Targeted fecal metabolomics in 35 patients with GLM and 26 healthy controls identified increased butanoic acid, but its modest discrimination does not establish diagnostic utility in the clinical differential (58). Higher isohexanoic acid levels were associated with recurrence among 34 patients followed for one year; however, only five recurrence events occurred, making the reported performance vulnerable to statistical instability (**Table 1**). These findings concern microbiota-derived metabolites and do not directly demonstrate metabolic reprogramming of lesional immune cells.

Epitranscriptomic regulation represents a less developed area in GLM biomarker research. Mechanistically, m6A modification can influence the stability, translation, and turnover of transcripts involved in cytokine signaling and immune-cell differentiation, thereby potentially contributing to the persistence of dysregulated inflammatory programs. However, whether such epitranscriptomic regulation operates directly in GLM lesions has not yet been demonstrated (59). Therefore, m6A-related signatures should be considered a future biomarker-discovery direction rather than established GLM biomarkers. Accordingly, the lack of direct GLM-specific evidence linking epigenetic remodeling or immune-cell metabolic reprogramming to persistent inflammatory states represents an important mechanistic gap that warrants investigation in future lesion-level multi-omics studies.

## 3 Differential diagnosis

Differential diagnosis is one of the most clinically relevant applications of biomarkers in GLM. Due to imaging similarities between GLM and breast cancer, accurate differentiation is challenging, making the combination of serum biomarkers an important strategy. Velidedeoglu et al. evaluated the fibrinogen-to-albumin (Fib/Alb) ratio in 55 patients with IGM, 50 with breast cancer, and 50 healthy controls (60). Its discrimination between IGM and breast cancer addresses a clinically relevant comparison, but performance was assessed within the study cohort without independent validation. Its additional value beyond clinical and imaging assessment therefore remains uncertain (**Table 1**). Zhou et al. compared 73 patients with GLM and 60 patients with benign breast tumors and found significantly higher serum IL-6 levels in the GLM group (61). However, this study primarily demonstrated group-level differences, and whether IL-6 provides additional discriminatory value beyond conventional clinical and pathological assessment remains unclear. Molecular biomarkers may provide additional discriminatory information. Aksan et al. found that serum miR-21 expression was significantly higher in breast cancer than in IGM, while PTEN levels also differed significantly between the two groups, suggesting potential adjunctive value for differential diagnosis (62). Ling et al. evaluated serum miR-451a, miR-5571-3p, CLN6, HSD11B1, and PDE4 in patients with mass-stage GLM, breast cancer, and fibroadenoma, with 55 patients per group (63). Combined miR-451a and miR-5571-3p achieved an apparent AUC of 1.00 for GLM versus the pooled breast cancer and fibroadenoma groups. However, this performance requires independent validation and does not establish pairwise discrimination between GLM and breast cancer or applicability to other disease stages (**Table 1**).

Serum cytokines have also been investigated for differential diagnosis. Yigitbasi et al. reported altered serum IL-33 and soluble ST2 (sST2) levels between patients with idiopathic granulomatous mastitis and breast cancer (64). In another study involving 54 patients with granulomatous mastitis and 58 patients with non-mass breast cancer, the combination of MRI perfusion imaging with serum SAA and TFF1 achieved an AUC of 0.935 and showed greater diagnostic performance than MRI, SAA, or TFF1 alone (65). Similarly, ultrasonography combined with serum sICAM-1 and IL-1β achieved an accuracy of 82.04%, with a sensitivity of 88.35% and specificity of 75.73% for distinguishing GLM from breast cancer (66). More recent ultrasonographic studies have further demonstrated the potential of conventional and contrast-enhanced ultrasound parameters for differentiating granulomatous mastitis from malignant breast lesions, while also providing information on treatment response and recurrence risk (67). Zeng et al. assessed multimodal ultrasonography and blood cell parameters in 57 patients with GLM and 100 with invasive ductal carcinoma (68). Although leukocyte counts differed between groups, the reported AUC of 0.965 corresponded to conventional ultrasonography combined with contrast-enhanced ultrasonography. It should therefore not be attributed to blood biomarkers or interpreted as evidence of their incremental diagnostic value.

Papila et al. evaluated serum α-defensin 1, β-defensin 1, and β-defensin 2 in 50 patients with IGM, 50 with breast cancer, and 50 healthy controls (69). Although defensin concentrations differed between groups, they were not retained as independent predictors in the final multivariable analysis; the reported AUC of 0.83 for breast cancer versus IGM belonged to a model containing CA15^–3^ and CA125. Thus, the independent diagnostic contribution of defensins remains uncertain.

Evidence for IL-33 is also inconsistent. Yigitbasi et al. suggested potential discriminatory value for serum IL-33 in differentiating IGM from breast cancer (64). However, Haghbin et al. found no significant difference between 25 patients with IGM and 28 with breast cancer (P = 0.43) (70). These findings do not support a consistently reproducible discriminatory role for serum IL-33.

Plasma cell mastitis (PCM) is a duct-centered inflammatory condition considered in the differential diagnosis of GLM (1). Their overlapping clinical presentations can make differentiation challenging. Kong et al. compared tissue specimens from patients with GLM (n = 36), PCM (n = 17), and breast cystic hyperplasia (n = 10). CD68, CD163, and CD57 expression did not differ significantly between GLM and PCM, and IgG4 expression was generally low in both groups (43). These findings do not establish the diagnostic utility of these markers for distinguishing GLM from PCM. In a separate study, Li et al. investigated differentiation between GLM and periductal mastitis (PDM). The terms PCM and PDM are retained here according to the respective studies rather than treated as interchangeable diagnostic labels. Li et al. developed a logistic regression model incorporating red cell distribution width, mean platelet volume, C4, IFN-γ, prothrombin time, and D-dimer in 60 patients with GLM and 60 with PDM. The reported AUC was 0.807 in the internal test cohort and 0.750 in a validation cohort comprising 14 patients with PDM and six with GLM from another campus of the same hospital (71). These findings suggest potential adjunctive diagnostic value, although the retrospective case-control design and small validation cohort limit generalizability.

## 4 Prognostic and disease-monitoring biomarkers

Beyond initial diagnosis, biomarkers may provide information about abscess-associated disease phenotypes. Du et al. developed a multivariable model incorporating neutrophil count, ESR, IL-4, IL-10, and IFN-α to distinguish patients with and without abscess-forming GLM (55). The combined model showed better discriminatory performance than the individual indicators. However, patients were classified according to the presence or absence of abscesses, and prospective prediction of incident abscess formation has not yet been established. Independent validation and prospective evaluation in patients without abscesses at baseline are therefore needed before the model can be used to predict future abscess development.

Routine inflammatory indices have been investigated for recurrence risk stratification, but their predictive performance remains incompletely established (24, 25). The NLR threshold of 5.02 was derived from a retrospective surgical cohort comprising 41 patients with IGM and eight recurrence events across two centers, without an independent validation cohort (72). Similarly, the association between SII >836 × 10^9^/L and recurrence was observed in 245 surgically treated patients with GM (24). These treatment-specific cohorts do not establish the transportability of either threshold to patients receiving other management strategies.

Ciftci et al. evaluated the albumin-to-globulin ratio (AGR) in 85 patients with IGM, including 16 with recurrence (73). A low AGR was associated with recurrence, whereas NLR and PLR did not differ significantly between recurrence groups. However, the AGR threshold was selected and evaluated in the same single-center retrospective cohort, and the limited event count and absence of independent validation restrict its clinical applicability (**Table 1**).

Multivariable recurrence models include a clinical nomogram developed in 287 patients with GLM and a subsequent multicenter machine-learning model (74, 75). Li et al. also developed a neural network using a single-center cohort of 212 patients with IGM, including 37 recurrence events (76). Its internal validation and test subsets each contained only six recurrence events. The reported discrimination therefore requires external confirmation, and the contribution of inflammatory biomarkers should be distinguished from that of clinical predictors (**Table 1**).

Endocrine biomarkers may provide complementary prognostic information. In a retrospective cohort of 130 patients, Huang and Wu found that changes in PRL levels before and after treatment were independently associated with GLM recurrence, with patients whose PRL increased after treatment showing a higher recurrence risk (28). More recently, elevated PRL has also been associated with larger lesion size and a lower 6-month cure rate, further supporting the potential value of serial PRL measurement for disease monitoring (29). Nevertheless, these findings remain observational, and the optimal timing and threshold for PRL-based risk stratification have not been established.

**Table 1** summarizes the study populations, outcome events, comparators, reported performance, and validation status of selected diagnostic and prognostic biomarkers and models. Comparisons against healthy controls are distinguished from disease-specific differential diagnosis, and internal evaluation is distinguished from independent validation.

## 5 Treatment

### 5.1 Biomarker-informed and targeted therapy

Microbiological biomarkers currently represent one of the clearest examples of biomarker-informed treatment in GLM. Identification of *Corynebacterium* species, together with antimicrobial susceptibility testing, may help select antimicrobial therapy in patients with microbiologically confirmed infection (77, 78). In particular, *Corynebacterium parakroppenstedtii*-associated GLM has been characterized by distinct antimicrobial susceptibility profiles, supporting pathogen-directed rather than empirical antibiotic selection (77). Nevertheless, the clinical benefit of antimicrobial therapy is likely to depend on microbiological evidence of infection, and antibiotics should not be regarded as a universally applicable treatment for GLM.

Current immunomodulatory treatment for GLM still relies primarily on glucocorticoids and conventional immunosuppressive agents such as methotrexate, for which clinical efficacy and tolerability have been evaluated in a recent systematic review and meta-analysis (79). However, no validated immune biomarker is currently available to prospectively identify which patients are most likely to benefit from glucocorticoids or methotrexate. Recent consensus recommendations have also emphasized treatment selection according to clinical classification, standardized criteria for treatment discontinuation, and structured follow-up in GLM (80).

The identification of increased TNF-α signaling in GLM has also raised interest in cytokine-targeted therapy. A recent retrospective cohort reported clinical effectiveness of TNF inhibitors in patients with IGM (81), suggesting a potential therapeutic role for TNF blockade in selected refractory cases. However, TNF-α concentration itself has not yet been validated as a predictive biomarker for treatment response, and prospective studies are needed to determine whether cytokine profiling can guide patient selection for TNF-inhibitor therapy.

Several biomarker-defined inflammatory pathways have also been explored as therapeutic targets in preclinical models. Ruxolitinib attenuated inflammatory responses through inhibition of JAK/STAT3 signaling in a rat model of granulomatous mastitis (82), while targeting the S100A8/S100A9 axis with paquinimod reduced NET formation and inflammatory injury in experimental GLM (41). These findings support the biological druggability of JAK/STAT3- and S100A8/S100A9-related pathways, but neither pathway has yet been validated as a treatment-selection biomarker in clinical GLM.

### 5.2 Traditional medicine and clinical molecular changes

Human studies in GM, GLM, or IGM have reported treatment-associated changes in inflammatory and immune markers following Zicao Yanghe Decoction, Chuang Ling Ye, Huaier granules combined with Yanghe Decoction, Yiqi Shugan Formula, Qingre Jiedu Formula, Yiqi Heying Formula and Chaiqin Xiaoyong Decoction (83–89). These findings remain exploratory and do not establish the measured markers as predictors of treatment response or tools for treatment selection. Network pharmacology provides computational predictions of potential therapeutic targets rather than biomarker evidence. Study populations, treatment comparisons, measured markers, and limitations are summarized in **Supplementary Table 2**.

## 6 Discussion

The clinical value of GLM-associated biomarkers depends on the decision they are intended to support. Current evidence most directly supports adjunctive assessment of inflammatory burden (20, 21) and microbiologically informed antibiotic selection (77, 78), whereas differential diagnosis, recurrence prediction, and immune-based treatment selection remain less established. Research priorities should therefore reflect clinical relevance, assay accessibility, and existing evidence (**Table 1**).

For differential diagnosis, the Fib/Alb ratio merits further evaluation because it is readily measurable and has been examined against breast cancer, while circulating miRNA panels warrant replication because they offer a distinct molecular approach (60, 62, 63). Neither is ready to replace histopathological confirmation. The next step should be a prospective study enrolling consecutive patients with diagnostically uncertain breast lesions, including malignant and inflammatory alternatives. Biomarkers should be measured before treatment and interpreted independently of the reference diagnosis. Prespecified models should be evaluated for their additional value beyond clinical and imaging assessment and subsequently tested in an independent cohort. Disease-specific comparisons are essential. Performance against pooled breast cancer and fibroadenoma groups does not establish separately validated discrimination against either condition. ISM1 and IL-33 currently merit lower priority for diagnostic translation because relevant disease controls or consistent discriminatory findings remain lacking (35, 64, 70).

For disease-activity assessment, CRP and ESR are practical priorities, with serial PRL measurement providing a complementary research focus in patients with endocrine abnormalities (20, 21, 29). Prospective longitudinal cohorts should test whether within-patient changes correspond to standardized clinical and ultrasonographic activity measures, precede deterioration, or improve decisions about treatment adjustment. Sampling should account for infection and treatment exposure. Abscess prediction requires a different design. The model incorporating neutrophil count, ESR, IL-4, IL-10, and IFN-α should be evaluated in patients without abscesses at baseline, with subsequent abscess formation defined as the outcome (55). This would distinguish prediction of a future complication from recognition of an existing disease phenotype.

For recurrence prediction, NLR, SII, and AGR should be prioritized for comparative validation because they are inexpensive and already supported by clinical association studies, although their proposed thresholds remain unconfirmed (24, 25, 72, 73). A multicenter cohort beginning at documented remission should use a uniform recurrence definition and account for disease extent, microbiological findings, endocrine status, and treatment strategy. Continuous marker values and prespecified thresholds should be compared within the same cohort. Candidate models require assessment of calibration and added clinical value, followed by independent validation. Complex machine-learning models should demonstrate improvement over a simple clinical model, while high discrimination in small internal subsets is insufficient to establish usefulness (74–76).

For treatment selection, pathogen identification and susceptibility testing provide a more direct decision pathway than circulating immune profiles (77, 78). Whether TNF-related or other immune signatures identify differential treatment benefit requires prospective comparative studies assessing the interaction between baseline biomarker status and treatment effect (81). Changes observed after therapy, including those reported in traditional medicine studies, may support pharmacodynamic investigation but do not establish a pretreatment selection biomarker. Establishing uniform criteria for evaluating clinical efficacy is essential to compare treatment effects across studies and to validate candidate biomarkers for treatment selection.

Mechanistic research should support these clinical priorities by linking lesional immune-cell states to reproducible, clinically measurable signals. Paired tissue and blood sampling across defined disease stages could establish whether candidate signatures reflect local inflammation and track relevant outcomes (9, 34). Standardized assays, explicit disease definitions, and independent replication are essential. The immediate translational priority is therefore validation of accessible markers for defined clinical questions, alongside focused development of molecular candidates with evidence beyond healthy-control comparisons.

## 7 Conclusion

This review summarizes current evidence on GLM-associated biomarkers and their potential applications in diagnosis, differential diagnosis, disease monitoring, recurrence prediction, and treatment guidance. Routine inflammatory and endocrine markers are currently the most clinically accessible, whereas emerging molecular signatures and predictive models remain largely exploratory and require further validation. Future multicenter prospective studies with standardized sampling and longitudinal follow-up are needed to translate promising biomarkers into clinically applicable tools.
