# Supplementary material for: Granulomatous lobular mastitis: diagnostic and prognostic biomarkers
Source: Front Immunol. 2026 Oct 22;17:1960862. doi: 10.3389/fimmu.2026.1960862 (PMC13735786; doi:10.3389/fimmu.2026.1960862)
Supplement: Supplementary file 1 [file Table1.docx]

Supplementary Material

# 1 Supplementary Figures and Tables

## 1.1 Supplementary Tables

| **Application** | **Biomarker** | **Specimen** | **Comparison/clinical setting** | **Main finding** | **Sample size** | **Ref.** |
| --- | --- | --- | --- | --- | --- | --- |
| **Diagnosis** | **IL-1β** | Serum | IGM vs HC | ↑ | 11 vs 7 | (21) |
|  |  | Serum | IGM vs HC | ↓ | 32 vs 18 | (56) |
|  | **IL-2** | Serum | IGM vs HC | ↑ | 11 vs 7 | (21) |
|  | **IL-4** | Serum | IGM vs HC | NS | 47 vs 30 | (51) |
|  | **IL-6** | Serum | IGM vs HC | ↑ | 11 vs 7 | (21) |
|  |  | Serum | GLM vs HC | ↑ | 30 vs 15 | (22) |
|  |  | Serum | IGM vs HC | NS | 32 vs 18 | (56) |
|  | **IL-8** | Serum | IGM vs HC | ↑ | 47 vs 30 | (51) |
|  |  | Serum | IGM vs HC | NS | 32 vs 18 | (56) |
|  | **IL-10** | Serum | GLM vs HC | ↑ | 30 vs 15 | (22) |
|  |  | Serum | IGM vs HC | ↑ | 47 vs 30 | (51) |
|  |  | Serum | IGM vs HC | ↓ | 32 vs 18 | (56) |
|  | **IL-12p70** | Serum | IGM vs HC | ↑ | 11 vs 7 | (21) |
|  |  | Serum | IGM vs HC | NS | 32 vs 18 | (56) |
|  | **IL-17 / IL-17A** | Serum | IGM vs HC | ↑ | 11 vs 7 | (21) |
|  |  | Serum | IGM vs HC | ↑ | 47 vs 30 | (51) |
|  |  | Serum | IGM vs HC | NS | 26 vs 15 | (53) |
|  |  | Serum | IGM vs HC | NS | 32 vs 18 | (56) |
|  | **IL-18** | Serum | IGM vs HC | ↓ | 32 vs 18 | (56) |
|  | **IL-22** | Serum | IGM vs HC | ↑ | 26 vs 15 | (53) |
|  | **IL-23** | Serum | IGM vs HC | ↑ | 26 vs 15 | (53) |
|  |  | Serum | IGM vs HC | NS | 32 vs 18 | (56) |
|  | **IL-33** | Serum | IGM vs HC | NS | 32 vs 18 | (56) |
|  | **IL-36α** | Serum | IGM vs HC | ↓ | 36 vs 37 | (32) |
|  | **TNF-α** | Serum | IGM vs HC | ↑ | 11 vs 7 | (21) |
|  |  | Serum | GLM vs HC | ↑ | 30 vs 15 | (22) |
|  |  | Serum | IGM vs HC | NS | 47 vs 30 | (51) |
|  |  | Serum | IGM vs HC | ↓ | 32 vs 18 | (56) |
|  | **IFN-α** | Serum | IGM vs HC | NS | 32 vs 18 | (56) |
|  | **IFN-γ** | Serum | IGM vs HC | NS | 32 vs 18 | (56) |
| **Differentiation** | **IL-1β** | Serum | GLM vs FA | NS (P = 0.129) | 107 vs 73 | (33) |
|  |  | Breast tissue | GLM vs normal tissue adjacent to FA | NS (P = 0.627) | 10 vs 10 | (33) |
|  |  | Serum | GLM vs benign breast tumors | NS | 73 vs 60 | (61) |
|  | **IL-6** | Serum | GLM vs FA | ↑ (P < 0.001) | 107 vs 73 | (33) |
|  | **IL-6** | Breast tissue | GLM vs normal tissue adjacent to FA | ↑ (P < 0.001) | 10 vs 10 | (33) |
|  |  | Serum | GLM vs benign breast tumors | ↑ | 73 vs 60 | (61) |
|  | **IL-8** | Serum | GLM vs FA | ↑ (P = 0.038) | 107 vs 73 | (33) |
|  | **IL-10** | Serum | GLM vs FA | NS (P = 0.624) | 107 vs 73 | (33) |
|  | **IL-17** | Breast tissue | GM vs normal tissue adjacent to benign breast lesions | ↑ (P < 0.001),  (positive expression 55.0% vs 20.0%) | 60 vs 60 | (54) |
|  | **IL-33** | Serum | IGM vs BC vs HC | IGM > BC > HC | 25 vs 32 vs 30 | (64) |
|  | **TNF-α** | Serum | GLM vs benign breast tumors | ↑ | 73 vs 60 | (61) |
|  | **IFN-γ** | Serum | GLM vs benign breast tumors | NS | 73 vs 60 | (61) |
| **Monitoring** | **IL-4** | Serum | Abscess vs non-abscess GLM | ↓ in the final multivariable model | 85 vs 41 | (55) |
|  |  | Breast tissue | Different GM stages | Stage-related variation with an inverse IL-2/IL-4 correlation | 60 total (20 per stage) | (57) |
|  | **IL-6** | Serum | Abscess vs non-abscess GLM | ↑ in univariate analysis only | 85 vs 41 | (55) |
|  | **IL-10** | Serum | Abscess vs non-abscess GLM | ↓ in the final multivariable model | 85 vs 41 | (55) |
|  |  | Serum | Active vs remission IGM | Disease-state-dependent difference; not consistently significant | 47 IGM total | (51) |
|  | **IL-17** | Serum | Abscess vs non-abscess GLM | ↓ in univariate analysis only | 85 vs 41 | (55) |
|  |  | Breast tissue | Mass vs abscess vs post-ulcerative GM | Positive expression 31.82%, 65.00%, 72.22% (P = 0.021) | 22 vs 20 vs 18 | (54) |
|  | **IL-2** | Breast tissue | Different GM stages | Stage-dependent difference; inversely correlated with IL-4 expression | 20/stage; 60 total | (57) |
|  | **IFN-α** | Serum | Abscess vs non-abscess GLM | ↓ in the final multivariable model | 85 vs 41 | (55) |

**Supplementary Table 1.** Study-specific cytokine findings by clinical application, specimen, and comparator in granulomatous mastitis.

Abbreviations: GLM, granulomatous lobular mastitis; IGM, idiopathic granulomatous mastitis; GM, granulomatous mastitis; HC, healthy controls; FA, fibroadenoma; BC, breast cancer; IL, interleukin; IFN, interferon; TNF, tumor necrosis factor; NS, no statistically significant difference. ↑ and ↓ indicate statistically significant increases and decreases, respectively. Reported findings are study-specific and should not be interpreted as uniform directional alterations across all GLM populations. Differences in comparator groups, biological specimens, disease stages, treatment status, and assay methods should be considered when interpreting between-study heterogeneity. For the abscess-prediction study by Du et al., IL-6 and IL-17 differed between abscess and non-abscess groups in univariate analyses, whereas IL-4, IL-10, and IFN-α were retained in the final multivariable model. Treatment-associated changes in measured markers do not establish their predictive value or clinical utility for treatment selection. Computationally predicted targets are not classified as measured biomarkers.

| **Treatment context** | **Biomarker/molecular target** | **Evidence basis** | **Potential role in treatment** | **Translational status** | **Ref.** |
| --- | --- | --- | --- | --- | --- |
| Antibiotic therapy | *Corynebacterium* species identification and antimicrobial susceptibility | Human microbiological studies with multi-center pathogen-resistance data | Guides antibiotic selection in confirmed infection | Clinically actionable | (77,78) |
| Endocrine-directed therapy | PRL, ESR, MHC-II+ T cells, Treg percentage, IL-4, IL-5 | Retrospective human study with PSM | Identifies elevated PRL and explores response monitoring | Exploratory response markers | (29) |
| Anti-inflammatory therapy | CRP, ESR and routine inflammatory markers | Human clinical cohorts | Supports inflammatory response monitoring | Nonspecific monitoring markers | (20,21) |
| TNF inhibition | TNF pathway | Retrospective IGM treatment cohort | Supports investigation of TNF inhibition | Clinical treatment evidence without a validated predictive marker | (81) |
| JAK/STAT-directed therapy | JAK/STAT3 pathway | Rat GM model treated with ruxolitinib | Candidate pathway for targeted treatment | Preclinical target | (82) |
| NET-directed therapy | S100A8/S100A9, PAD4 and NET axis | Experimental GLM models with pharmacological inhibition | Candidate target for reducing NET formation | Preclinical target | (41) |
| Inflammasome/pyroptosis-related mechanisms | Caspase-1–GSDMD pyroptosis pathway | Human GLM tissue study | Identifies tissue pyroptosis pathways | Exploratory tissue markers with therapeutic and pharmacodynamic utility remains unvalidated | (39) |
| Macrophage-related mechanistic targets | CD86/CD206-associated phenotypes | Human case-control and single-cell studies | Characterizes macrophage phenotypes | Exploratory mechanistic markers | (33,34) |
| Topical Zicao Yanghe Decoction combined with dexamethasone ointment | NLRP3, caspase-1, and GSDMD | Randomized study of 40 GLM patients. Comparator: dexamethasone ointment alone | Assessment of changes in pyroptosis-related proteins | Small clinical study. Predictive value not assessed. | (83) |
| Topical Chuang Ling Ye | Local IL-1β, IFN-γ, and TNF-α | Randomized trial of 40 IGM patients. Comparator: control wound care | Assessment of changes in local inflammation | Small trial with four-week observation | (89) |
| Huaier granules combined with modified Yanghe Decoction | CRP, IL-2, IL-6, and CD4⁺/CD8⁺ ratio | Randomized study of 40 patients with mass-stage GLM. Comparator: modified Yanghe Decoction alone | Assessment of inflammatory and immune changes | Small study assessing the added effect of Huaier | (85) |
| Yiqi Shugan Formula combined with minimally invasive debridement | WBC count, IL-6, and CRP | Randomized study of 80 patients with abscess-stage GLM. Comparator: debridement alone | Assessment of changes in systemic inflammation | Single-center combination-treatment study | (86) |
| Qingre Jiedu Formula | WBC count, neutrophil count, CRP, and ESR | Randomized study of 60 acute GM patients. Comparator: cefuroxime axetil | Assessment of short-term changes | Seven-day treatment and seven-day follow-up. | (87) |
| Chaiqin Xiaoyong Decoction | TNF-α, IL-1β, IL-6, PPAR-γ, NF-κB p65, and macrophage-related markers | Before and after tissue study of six mass-stage GLM patients with six normal breast tissue controls | Assessment of tissue and macrophage-related changes | Small study without an untreated GLM group | (88) |

**Supplementary Table 2.** Measured treatment-associated biomarkers and mechanistic therapeutic targets in granulomatous mastitis.

Abbreviations: GLM, granulomatous lobular mastitis; IGM, idiopathic granulomatous mastitis; PRL, prolactin; CRP, C-reactive protein; ESR, erythrocyte sedimentation rate; TNF, tumor necrosis factor; JAK, Janus kinase; STAT, signal transducer and activator of transcription; NETs, neutrophil extracellular traps; NLRP3, NOD-like receptor family pyrin domain containing 3; GSDMD, gasdermin D; MHC II, major histocompatibility complex class II; GM, granulomatous mastitis; PSM, propensity score matching; PAD4, peptidylarginine deiminase 4. Clinically actionable indicates that biomarker information can directly support a treatment decision in current clinical practice. Pharmacodynamic candidate refers to a molecular or immune parameter that changes in association with treatment but has not been prospectively validated as a predictor of therapeutic response. Mechanistic targets identified primarily in animal, in vitro, or computational studies should not be interpreted as validated treatment-selection biomarkers.
